# NLRP3 inflammasome activation contributes to *Listeria monocytogenes*-induced animal pregnancy failure

**DOI:** 10.1186/s12917-016-0655-2

**Published:** 2016-02-24

**Authors:** Wenyan Li, Yumei Chang, Shuang Liang, Zhenyu Zhong, Xiujin Li, Jiexia Wen, Yonghong Zhang, Jianlou Zhang, Liyue Wang, Hongyu Lin, Xuebin Cao, Heling Huang, Fei Zhong

**Affiliations:** Laboratory of Molecular Virology and Immunology, College of Veterinary Medicine, Agricultural University of Hebei; Hebei Engineering and Technology Research Center of Veterinary Biotechnology, Baoding, 071000 China; Department of Gynaecology and Obstetrics, 252 Hospital of Chinese PLA, Baoding, 071000 China; Department of Medicine, School of Medicine, University of California San Diego, La Jolla, CA 92093 USA; Department of Pharmacology, School of Medicine, University of California San Diego, La Jolla, CA 92093 USA; Department of Biotechnology, College of Environmental and Chemical Engineering, Yanshan University, Qinhuangdao, 066004 China; Department of Biology, School of Medicine, Hebei University, Baoding, 071000 China

**Keywords:** *Listeria monocytogenes*, Listeriolysin O, Pregnancy failure, Inflammasome, NLRP3

## Abstract

**Background:**

Li*steria monocytogenes* (LM), a foodborne pathogen, can cause pregnancy failure in animals, especially in ruminants. Recent studies have shown that LM activates inflammasomes to induce IL-1β release in macrophages, however, whether the inflammasome activation regulates LM-induced pregnancy failure remains largely unknown. Here we used mouse model to investigate the molecular mechanism by which LM-induced inflammsome activation contributes to LM-associated pregnancy failure

**Results:**

We showed that wild-type, but not Listeriolysin O-deficient (Δ*hly*) LM, significantly reduced mouse embryo survival, accompanied by the increase of IL-1β release and caspase-1 activation. IL-1β neutralization significantly reduced the LM-induced embryo losses, suggesting that LM-induced pregnancy failure was associated with LLO-induced inflammasome activation. To dissect the inflammasome sensor and components responsible for LM-induced caspase-1 activation and IL-1β production, we used wild-type and NLRP3^−/−^, AIM2^−/−^, NLRC4^−/−^, ASC^−/−^, caspase-1^−/−^ and cathepsin B^−/−^ mouse macrophages to test the roles of these molecules in LM-induce IL-1β production. We found that NLRP3 inflammasome was the main pathway in LM-induced caspase-1 activation and IL-1β production. To explore the mechanism of LM-induced pregnancy failure, we investigated the effects of LM-infected macrophages on SM9-1 mouse trophoblasts. We found that the conditioned medium from LM-infected-macrophage or the recombinant IL-1β significantly up-regulated TNFα, IL-6 and IL-8 productions in trophoblasts, suggesting that the LM-induced macrophage inflammasome activation increased trophoblast pro-inflammatory cytokine production, which was adverse to the animal pregnancy maintenance.

**Conclusions:**

Our data demonstrated that the LLO-induced NLRP3 inflammasome activation plays a key role in LM-induced pregnancy failure, and inflammasome-mediated macrophage dysregulation on trophoblasts might be involved in the pregnancy failure.

## Background

*Listeria monocytogenes* (LM), a gram-positive bacterium which can grow and replicate in the cytosol of the host cells, is a food-borne zoonotic pathogen. They can cause meningitis, septic gastroenteritis, spontaneous abortion, premature labor and stillbirth in humans and animals, especially in ruminants such as cattle, goat and sheep [1–3]. LM can infect placental and fetal tissues by crossing intestinal and placental barriers, resulting in abortion, premature birth or stillbirth [4]. During infection, LM is engulfed by the placental macrophages via phagocytosis and produces listeriolysin O (LLO) and other pathogenic toxin, resulting in endo-lysosome rupture, therefore, LM has the opportunity to escape from lysosome into cytosol, where they can rapidly replicate, leading to cell damage during the later stages of infection [5].

Infectious pathogens can be recognized by host immune system through the pattern recognition receptors (PRRs) on monocytes/macrophages and dendritic cells, including membrane-bound receptors and intracellular receptors. TOLL-like receptors (TLRs) are examples of membrane-bound PRRs which recognize the extracellular pathogens and trigger NF-κB-mediated proinflammatory cytokine expression (mainly including pro-IL-1β, TNFα and IL-6) and interferon regulatory factor (IRF)-mediated type I interferon production [6]. The intracellular PRRs mainly include NOD-like receptors (NLRs), RIG-I-like receptors (RLRs) and AIM2-like receptors (ALRs) which recognize intracellular pathogens as well as endogenous danger signals (ATP, ROS, K^+^ efflux, Ca^++^ influx or lysosome damage) [7–9]. It is well established that upon recognition of intracellular pathogens or endogenous danger signals, some NLRs can assemble into a multiprotein complex called the inflammasome which can activate caspase-1. The activated caspase-1 then cleaves pro-IL-1β and pro-IL-18 into bioactive forms [10, 11]. Moreover, the activated caspase-1 also triggers a rapid, caspase-1-dependent cell death, termed pyroptosis [12].

Although LM was identified to cause animal spontaneous abortion and pregnancy disorder, the underlying mechanism is still largely unknown. It has been shown that pregnancy failure is largely associated with immune disorders caused by pathogen infection or tissue inflammation [13, 14]. In placenta tissues, there are many immune cells, including macrophages and natural killer cells, playing pivotal roles in defending the host against pathogens and maintaining normal immune homeostasis [15]. Trophoblasts at the maternal/fetal interface also play a key role in maintaining fetal development and local immune balance by cross-talk with adjacent cells, especially macrophages [16]. For example, the trophoblasts can induce recruitment and differentiation of monocytes/macrophages and the latter induce pro-inflammatory cytokine and chemokine production to support trophoblast growth and survival [17, 18]. However, how LM infection affects the innate immune response of macrophages and how LM-infected macrophages regulate the trophoblasts have not been fully investigated. It was reported that LM can activate membrane-bound TLR2 and TLR5 mediating pro-inflammatory cytokine productions, including pro-IL-1β [19, 20]. Recent reports have shown that LM can activate intracellular NLRs (namely NLRP3 and NLRC4) and ALRs (such as AIM2) mediating inflammasome activation, leading to the IL-1β release from macrophages [21–23]. Increased IL-1β production has been considered an important contributor for animal spontaneous abortion. We therefore speculated that the inflammasome activation might play a role in LM-induced pregnancy failure. So far four inflammasome receptors have been identified involved in LM-induced inflammasome activation, NACHT-, LRR- and PYD-containing Protein 3 (NLRP3), absent in melanoma-2 (AIM2), NLR family CARD domain-containing protein 4 (NLRC4), retinoic acid inducible gene 1 (RIG-I), respectively activated by LLO, bacterial DNA, flagella and released DNA or RNA, respectively [21–24]. However, which is the dominant receptor in LM-induced inflammasome activation is still unclear. Whether inflammasome activation in macrophages plays the crucial roles in LM-induced animal pregnancy failure is unknown. In this study, we first tested whether LM-induced inflammasome activation is associated with pregnancy failure *in vivo*. Then we further determined which inflammasome receptor is predominant in LM-induced inflammasome activation. Finally we analyzed the regulation of LM-infected macrophages on trophoblasts in immune response to gain further insights of crosstalk between macrophages and trophoblasts during pregnancy, and explore the molecular mechanism underlying LM-induced immune disorders in maternal/fetal interface.

## Methods

### Mice, bacteria and cells

The C57B1/6 mice were purchased from Beijing Laboratory Animal Research Center. LM (serovar 1/2a strain EGD) and its LLO-deficient (Δ*hly*) LM derivative were the kind presents of Dr. Masao Mitsuyama, Kyoto University [25]. The mouse trophoblast cell line SM9-1 was kindly provided by Dr. Joan S. Hunt, University of Kansas [26]; Mouse bone marrow-derived macrophages originally isolated from C57Bl/6 mice (refereed to B6 cells hereafter) and its derived gene-knockout cells NLRP3^−/−^, AIM2^−/−^, NLRC4^−/−^, ASC^−/−^, caspase-1^−/−^ and cathepsin B^−/−^ cells, were kindly provided by Dr. Katherine Fitzgerald, University of Massachusetts [27]. This study was performed according to the recommendations in the Guide for the Care and Use of Laboratory Animals of the Agricultural University of Hebei. The protocol was approved by the Ethical Committee for Animal Research of the Agricultural University of Hebei (Permit Number: 16–2012) in 2005.

### LM amplification, infection and tissue preparation

LM and Δ*hly* LM were grown in brain heart infusion broth medium for amplification. The C57B1/6 female and male mice were kept in open top-wire cages under a 12 h-light/12 h-dark cycle with food and water *ad libitum*. Successful mating was confirmed by visual identification of vaginal plug formation. Pregnant mice were randomly divided into 7 groups of 8 mice. On gestation day 6, groups 2, 3 and 4 were infected with 5 × 10^4^, 5 × 10^5^ and 5 × 10^6^ Colony Forming Units LM/ mouse in PBS by tail intravenous injection, respectively; groups 5, 6, and 7 were infected with Δ*hly* LM at the same doses as above. Group 1 mice were treated with PBS as a control. On gestation day 9, blood samples were collected by orbital puncture and serum was separated. The mice were euthanized by CO_2_ inhalation, and embryo survival rates (ESR) were calculated according to the following formula: ESR = survived embryo number / (survived embryo number + resorbed embryo number). Placental tissues were isolated, minced with a sterile scalpel and homogenized in a glass homogenizer in ice-cold PBS containing 0.5 mM Phenylmethanesulfonyl fluoride (PMSF, protease inhibitor) (Invitrogen). The homogenate was centrifuged at 10,000 × g for 15 min and the supernatant was collected for further analysis on cytokines or proteins.

### Macrophage culture, infection and sample preparation

Mouse B6 macrophages cultured in 6-well plates in DMEM complete medium at a cell density of 2 × 10^6^ cells/well were infected with LM at the different multiplicity of infection (MOI) (MOI = 5, 10, 20, 40). Fresh DMEM containing 50 μg/mL gentamicin was added at 1 h post infection and culture was continued for 5 h [23]. The culture medium was then collected for cytokine measurements. The infected cells were lysed with lysis buffer (5 mM Tris–HCl, 25 mM KCl, 2 mM EGTA, 2 mM EDTA, 1 % NP-40, 15 mM NaCl and protease inhibitor) for Western blot assay. Total RNA were prepared with Trizol reagent (Invitrogen) for RT-PCR assay.

### Trophoblast culture and treatment

Trophoblast cell line SM9-1 cells were cultured in RPMI-1640 complete medium. The cells were seeded at a density of 2 × 10^6^ cells/well to T25 flasks (Corning) in 5 mL X-VIVO-5 medium (Lonza) and stimulated with 20 % culture medium of LM-infected macrophages or 10 ng/mL recombinant mouse IL-1β. After 24 h, the trophoblasts were collected for cytokine determination with RT-PCR and ELISA.

### ELISA and western blot

IL-1β concentration in cell culture medium, mouse serum or placental tissue extract was measured by ELISA, and caspase-1, NLRP3 and ASC were determined by Western blot as described previously [8]. ELISA assays for mouse TNFα, IL-6 and IL-8 were performed with commercial kits (R&D System) following the manufacturers’ instructions.

### RT-PCR

Total RNA of the cells was extracted with Trizol reagent and reverse transcription was performed using the EasyScript First-Strand cDNA Synthesis SuperMix kit (TransGen) following the manufacturer’s instructions. The specific DNA fragments were amplified by PCR using specific primers listed in Table 1.

**Table 1:** Primers for amplifying mouse cytokine cDNA by RT-PCR

Gene	Forward primer sequence (5′ → 3′)	Reverse primer sequence (5′ → 3′)	Accession No.	Size/bp
GAPDH	GAAGGGTGGAGCCAAAAGGGTCAT	CATTGGGGGTAGGAACACGGAAGG	GU214026.1	360
IL-1β	TACAGGCTCCGAGATGAACAACAA	TGGGGAACTCTGCAGACTCAAACT	BC011437.1	332
TNFα	AGGGGCCACCACGCTCTTCTGT	GCAAATCGGCTGACGGTGTGG	BC117057.1	358
IL-6	AACCACGGCCTTCCCTACTTC	TCTGGCTTTGTCTTTCTTGTTATC	NM_031168.1	383
IL-8	ATGGCTGCTCAAGGCTGGTC	AGGCTTTTCATGCTCAACACTAT	NM_011339.2	386
MCP-1	AGGTCCCTGTCATGCTTCTGG	TTACGGGTCAACTTCACATTCAAA	BC145867.1	367

### Intracellular cathepsin B detection

Intracellular cathepsin B either in lysosomes or in cytosol was detected by DQ Ovalbumin fluorescence [28, 29]. B6 cells were seeded on coverslips in 24-well plates and infected with LM for 6 h or treated with CA-074-Me, a cathepsin B inhibitor (Santa Cruz) for 12 h. The cells were fixed in 4 % PFA for 15 min and incubated with 647-conjugated cholerotoxin B (Life Technologies) for 60 min in the dark for cell membrane staining (red). Cells were then permeabilized by 0.2 % saponin for 20 min and stained with 0.2 mg/mL DQ Ovalbumin, cathepsin B substrate (green) (Life Technologies) and 1 μg/mL Hoechst 33342 for nuclear staining (blue) (Life Technologies) containing 0.2 % saponin for 30 min. After washing with PBS, the cells were imaged immediately by laser scanning confocal microscopy.

### Cytosolic cathepsin B measurement

Cytosolic cathepsin B of LM-infected B6 cells was measured using ELISA Kit (Abcam). LM-infected B6 cells were washed with PBS on ice and permeabilized with 25 μg/mL digitonin/ 1 mM PMSF in PBS for 15 min at 4 °C to lyse the plasma membrane while leaving the intracellular membranes intact. During this procedure, cathepsin B released from lysosomes in the cytosol is further released into the suspending medium. Cathepsin B levels in the medium therefore reflect the cytosolic levels [30].

### Statistical analysis

The significance of differences between groups was evaluated by one-way analysis of variance (ANOVA) with Dunnett’s post-comparison test for multiple groups to control group, or by Student’s *t*-test for two groups. R^2^ was calculated from the Pearson correlation coefficient. Analyses were performed with Prism software (Graph Pad Inc.).

## Results

### Inflammasome activation by LM correlates with a reduction in mouse embryo survival *in vivo*

**Fig. 1:**
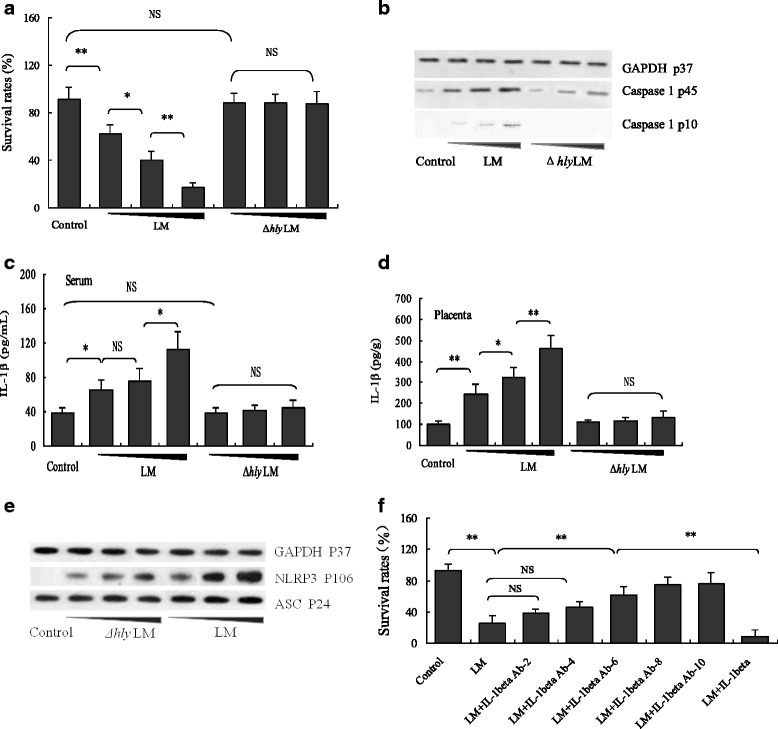
Effects of LM infection on embryo survival, caspase-1 expression/activation and IL-1β production in pregnant mice (**a**) Embryo survival rates of pregnant mice (*n =* 8) following infection with LM or Δ*hly* LM at different doses (5 × 10^4^, 5 × 10^5^ and 5 × 10^6^ CFU LM/ mouse in 0.2 mL PBS). **b** Pro-caspase-1 (P45) and activated caspase-1 (P10) levels in placental tissues detected by Western blot. **c** IL-1β levels measured by ELISA in the sera of pregnant mice infected with LM or Δ*hly* LM at the same doses as above. **d** IL-1β levels (including pro-IL-1β and mature IL-1β) detected by ELISA in the placenta of LM- and Δ*hly* LM-infected mice. **e** NLRP3 and ASC expressions in placenta of LM- and Δ*hly* LM-infected mice analyzed by Western blot. **f** The effects of IL-1β antibody on embryo survival rates of LM-infected pregnant mice. The pregnancy mice were treated with different amount of mouse IL-1β antibody (0, 2, 4, 6, 8, 10 μg/d) for 3 days, and infected with LM on the 2nd day of antibody treatment. The data are presented as means ± standard deviation. * *p <* 0.05; ** *p <* 0.01. NS: no significance

It has been shown that LM infection can cause animal pregnancy failure *in vivo* [12–14] and activate macrophage inflammasome in vitro [21–24]*.* However, whether LM-induced inflammasome activation is associated with LM-induced animal pregnancy failure is not clear. Our results showed that the ESR of the wild-type LM-infected mice was significantly lower than that of Δ*hly* LM-infected mice (Fig. 1a). Wild-type LM-induced mouse pregnancy failure accompanied with caspase-1 activation (Fig. 1b) in placental tissues and high-level IL-1β production in both sera (Fig. 1c) and placental tissues (Fig. 1d), suggesting that LM can activate inflammasomes by LLO *in vivo* and LM-induced animal pregnancy failure may associated with LM-mediated inflammasome activation. To further confirm this assumption, we also analyzed the effects of LM on NLRP3 and ASC expressions (inflammasome-related proteins) and showed that LM, either wild-type or Δ*hly* LM, could induce NLRP3, but not ASC expression in placental tissues (Fig. 1e) since ASC is a constitutively-expressed protein. In order to determine whether inflammasome-mediated IL-1β production contributes to LM-induced animal pregnancy failure, we used mouse IL-1β antibody to neutralize the IL-1β induced by LM in pregnant mice, and showed that high-level neutralizing antibody (more than 6 μg/d) significantly inhibits LM-induced mouse embryo loss (Fig. 1f), indicating that LM-induced inflammasome activation/IL-1β release was involved in animal pregnancy failure.

### LLO mediates the LM-induced inflammasome activation in mouse macrophages

**Fig. 2:**
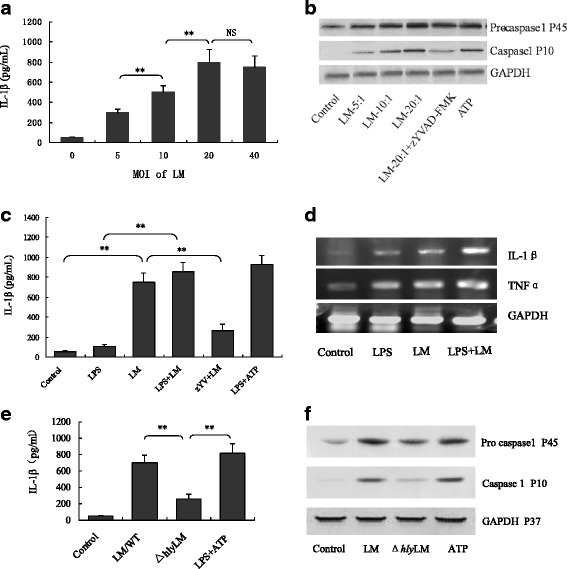
LM activates inflammasomes and stimulates pro-inflammatory cytokine production of mouse B6 macrophages (**a**) IL-1β concentrations in the media of B6 macrophages infected by LM at different MOI (5, 10, 20 and 40). **b** Western blot detection of pro-caspase-1 and activated caspase-1 in B6 macrophages following different treatments. **c** IL-1β concentrations in the culture media of B6 macrophages infected by LM (MOI = 20) and treated with different stimuli, including LM/zYV (zYVAD-FMK) (10 μM) as a negative control and LPS/ATP (0.5 μg/mL/5 mM) as a positive control for inflammasome activation. **d** Pro-IL-1β and TNFα detection in macrophages by RT-PCR. **e** IL-1β concentrations in the media of B6 macrophages infected with wild-type LM and Δ*hly* LM (MOI = 20). **f** Western blot detection of pro-caspase-1 and its active form in infected macrophages infected with wild-type LM and Δ*hly* LM (MOI = 20). The data in (**a**), (**c**) and (**e**) are shown as means ± standard deviation, and representative of three independent experiments. * *p <* 0.05; ** *p <* 0.01. NS: no significance

The above *in vivo* experiments suggested that LM could activate inflammasomes by LLO, which is consistent with a previous report [21]. To further confirm LM-induced inflammasome activation, we infected the immortalized bone marrow-derived macrophages originally isolated from C57BL/6 J mice (hereafter named B6 cells) with LM for 6 h and showed that LM significantly induced IL-1β release in a dose-dependent manner (Fig. 2a) and promoted caspapse-1 activation (Fig. 2b). The caspase-1 inhibitor zYVAD-FMK significantly blocked LM-induced IL-1β release (Fig. 2c), indicating LM can activate mouse B6 macrophage inflammasomes. Furthermore, LM also induced pro-IL-1β and TNFα expressions (Fig. 2d), suggesting LM *per se* can provide both signals that are required for bioactive IL-1β production. To explore whether LLO plays a key role in activating inflammasome, we infected macrophages *in vitro* with wild-type and Δ*hly* LM separately and analyzed IL-1β release and caspase-1 activation. Results showed that wild-type LM induced more IL-1β release (Fig. 2e) and caspase-1 activation (Fig. 2f) compared with Δ*hly* LM, indicating that LLO is the dominant determinant responsible for LM-induced inflammasome activation, although other stimuli, such as bacterial DNA and flagella may also partially contribute to the LM-induced inflammasome activation [22, 23].

### LLO activates inflammasome by endo-lysosome rupture and cathepsin B release

**Fig. 3:**
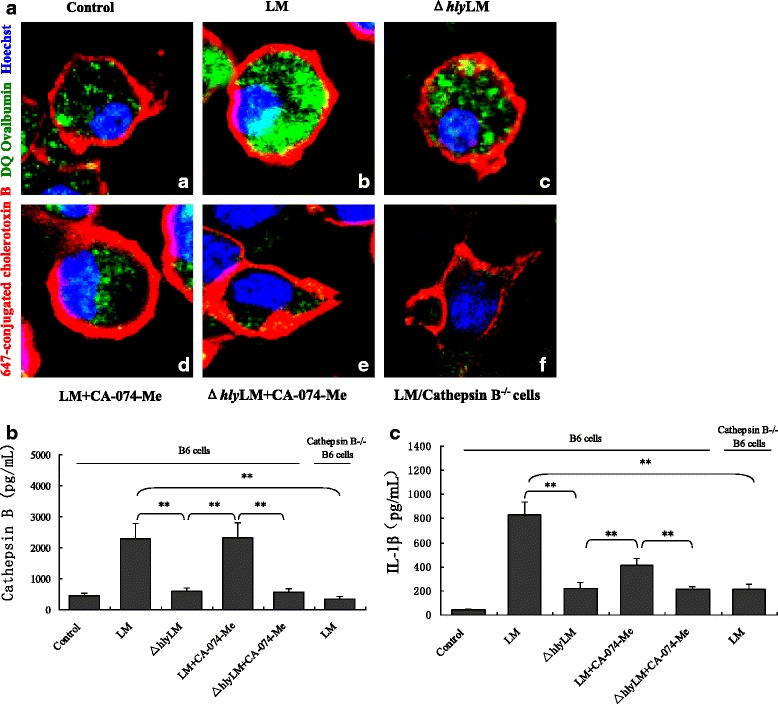
LM-induced lysosome damage and cathepsin B release from lysosomes in mouse B6 macrophage. **a** Confocal imaging of cathepsin B staining with DQ ovalbumin (green) in mouse B6 macrophages and cathepsin B^−/−^ B6 macrophages infected with LM or Δ*hly* LM (MOI = 20), with or without cathepsin B inhibitor CA-074-Me (The red color presented membrane and blue color presented nuclear): (a) untreated B6 cells; (b) LM-infected B6 cells; (c) Δ*hly* LM-infected B6 cells; (d) and (e) LM- and Δ*hly* LM-infected B6 cells pretreated with CA-074-Me (10 μM); (f) LM-infected cathepsin B^−/−^ B6 cells. **b** and **c** cathepsin B in the cytosol and IL-1β in the culture medium of LM- and Δ*hly* LM-infected B6 cells and cathepsin B^−/−^ B6 cells following different treatments. The data in **b** and **c** are represented as means ± standard deviation from three independent experiments. * *p <* 0.05; ** *p <* 0.01. NS: no significance

Damage to endo-lysosomes resulting in release of cathepsin B into the cytosol is considered one of the important activators of NLRP3 inflammasomes [28]. To test whether LM infection results in endo-lysosome damage and subsequent cathepsin B release, and whether LLO-induced inflammasome activation involved these processes, we infected wild-type and cathepsin B^−/−^ B6 cells with LM and Δ*hly* LM, respectively, and analyzed the distribution of cathepsin B in the cells by fluorescent staining. Results showed that high levels of cathepsin B (Fig. 3ab, b) and IL-1β (Fig. 3c) were present in the cytosol and culture medium, respectively, in wild-type- but not Δ*hly* LM-infected B6 cells (Fig. 3ac, b), indicating that LM-caused lysosome damage/cathepsin B release was dependent on LLO. As expected, no cathepsin B was detected in the cytosol or other cellular compartments of wild-type LM-infected cathepsin B^−/−^ B6 cells (Fig. 3af). Furthermore, CA-074-Me significantly reduced cytosolic cathepsin B fluorescent signal from wild-type LM-infected B6 cells (Fig. 3ad). Importantly, the IL-1β and cathepsin B levels in the culture medium of LM-infected cathepsin B^−/−^ B6 were both greatly decreased compared with that in LM-infected B6 cells (Fig. 3b and c), suggesting that cathepsin B is a critical player for LM-induced inflammasome activation. Together, these results indicate that LM induces rupture of endo-lysosome membrane by LLO, leading to cytosolic cathepsin B release which subsequently activates inflammasome [28].

### LM dominantly activates NLRP3 inflammasome by LLO in mouse macrophages

**Fig. 4:**
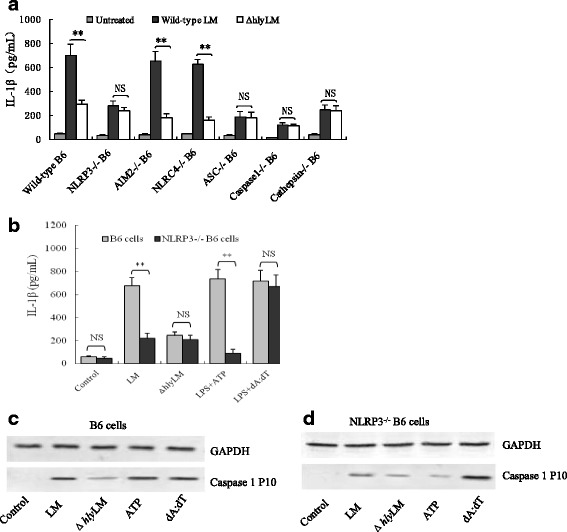
LM activates NLRP3 inflammasome by LLO in mouse B6 macrophages (**a**) IL-1β levels in the culture medium of mouse B6 cells and its derived gene knock-out cells infected with wild-type or Δ*hly* LM was measured by ELISA. **b** IL-1β levels in the culture medium of wild-type and NLRP3^−/−^ B6 cells infected with wild-type LM and Δ*hly* LM, respectively. **c** and (**d**) Western blot for detection of activated caspase-1 (P10) in wild-type and NLRP3^−/−^ B6 cells infected with LM or Δ*hly* LM. LPS + ATP as a positive control of NLRP3 inflammasome activation. LPS + dA:dT (1.5 μg/10^6^ cells) as a positive control of AIM2 inflammasome activation. The data in (**a**) and (**b**) are shown as means ± standard deviation from three independent experiments. * *p <* 0.05; ** *p <* 0.01. NS: no significance

To further delineate the molecular mechanism underlying LM-induced inflammasome activation, we used gene knockout B6 cells (NLRP3^−/−^, AIM2^−/−^, NLRC4^−/−^, ASC^−/−^, caspase-1^−/−^ and cathepsin B^−/−^) to determine which genes are critical for LM-induced IL-1β release. We found that wild-type LM, but not Δ*hly* LM, significantly increased IL-1β release from wild-type, AIM2^−/−^ and NLRC4^−/−^ B6 cells, but failed to do so in NLRP3^−/−^, ASC^−/−^, caspase-1^−/−^ and cathepsin B^−/−^ B6 cells (Fig. 4a). These data indicate that LM mainly activated NLRP3 inflammasome by LLO-induced cathepsin B release. To further confirm LM mainly activates NLRP3 inflammasome, we used wild-type and NLRP3^−/−^ B6 cells to analyze the IL-1β release and caspase-1 activation upon LM infection (ATP and poly (dA:dT) were used as the NLRP3 and AIM2 inflammasome activators, respectively). Results showed that the deficiency in NLRP3 dramatically reduced LM-induced IL-1β release and caspase-1 activation and these effects are LLO dependent as Δ*hly* LM-infected cells had diminished IL-1β release (Fig. 4b-d). These results collectively indicates that LM dominantly activates NLRP3 inflammasome.

### LM-induced IL-1β release in macrophages influences trophoblasts in immune responses

**Fig. 5:**
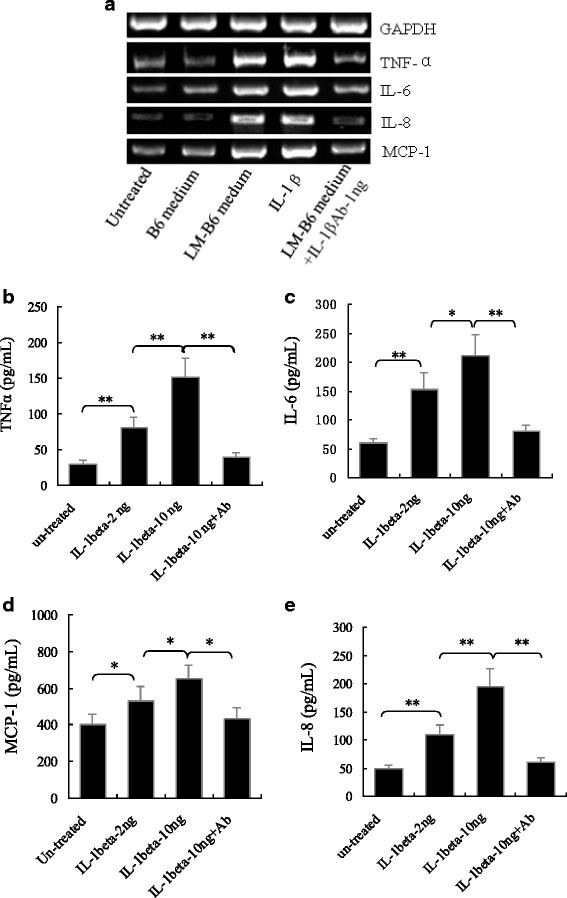
Levels of TNFα, IL-6, IL-8 and MCP-1 in mouse trophoblasts (SM9-1) and their media treated with LM-infected mouse B6 macrophage secretory products or IL-1β. **a** mRNA levels of TNFα, IL-6, IL-8 and MCP-1 detected by RT-PCR in trophoblasts treated with the secretory products of LM-infected macrophages. **b**-**e** Protein levels of TNFα, IL-6, IL-8 and MCP-1 in the medium of trophoblasts treated with IL-1β (2, 10 ng/mL) and IL-1β (10 ng/mL) + its antibody (50 ng/mL). The data in (B-E) are shown as means ± standard deviation from three independent experiments. * *p <* 0.05; ** *p <* 0.01. NS: no significance

Mutual communication between macrophages and trophoblasts in the maternal/fetal interface plays an important role in maintaining fetal development and local immune balance [17, 18]. To investigate whether LM-infected macrophage regulates trophoblasts, we performed RT-PCR to measure the expression levels of the inflammation-related cytokines and monocyte chemotactic protein-1 (MCP-1) in the trophoblasts upon treatment with either LM-conditioned macrophage medium (filtrated by 0.2 μm membrane) or recombinant IL-1β. As shown in Fig. 5a, both LM-conditioned macrophage medium and recombinant IL-1β could significantly induced TNFα, IL-6, IL-8 and MCP-1 expressions, IL-1β antibody significantly inhibited their induction. These results indicated that the LM-infected macrophages could regulate trophoblasts in inflammatory response via inflammasome/IL-1β pathway. To further confirm these results, we analyzed the levels of the three cytokines and MCP-1 by ELISA in the culture medium of trophoblasts treated with the recombinant IL-1β. It can be seen from Fig. 5b-e that IL-1β treatment significantly increased TNFα, IL-6, IL-8 and MCP-1 levels in trophoblast culture media in a dose-dependent manner. The high levels of the proiinflammatory cytokines would stimulate the local inflammation, and elevated MCP-1 might recruit inflammatory cells to the maternal/fetal interface to further exacerbate this response. None of these events would be conducive to proper embryonic development.

## Discussion

In this study, we demonstrated that LM-induced inflammasome activation positively correlated with mouse pregnancy failure. To the best of our knowledge, this is the first study linking LM-induced animal pregnancy failure with inflammasome activation. Previous studies on LM-induced pregnancy failure mainly focused on immune imbalance and trophoblast apoptosis induced by proinflammatory cytokine overexpression, anti-apoptosis substance (HO-1 and Bcl-XL) suppression [13, 14, 31]. In fact, inflammatory response has been identified to plays a crucial role in pathogen-induced pregnancy failure. It has been reported that alteration of IL-1β in maternal-fetal interface can cause experimental dysgenesis and abortion in mice [32, 33]. Our previous study revealed that *E. coli* heat-labile enterotoxin could activate NLRP3 inflammasome/IL-1β release, resulting in mouse embryo loss. In this study, we found that LM-induced pregnancy failure was closely associated with inflammasome activation based on LM-induced IL-1β overproduction and caspase-1 activation.

As noted above, four inflammasome receptors have been identified to be involved in LM-induced inflammasome activation [21–24], however, which was the dominant one was unclear. Our results first clarified that NLRP3 inflammasome is a dominant one activated by LM, which was mainly related to LLO.

During pregnancy, the communication and interaction between trophoblasts and macrophages in the maternal/fetal interface play an important role in embryo implantation, blood vessel formation, anti-pathogen defense and embryo development [17, 18]. Many studies have reported the role of trophoblast in regulating macrophage functions. For instance, trophoblast can secrete MCP1 to induce monocyte/macrophage recruitment [17, 18]. However, little is known about the regulation in the opposite direction. In other words, whether and how macrophage regulates trophoblast function at the maternal/fetal interface remains elusive. In this study, we treated trophoblasts with the secretory products of LM-infected macrophages and found that the proinflammatory cytokine (TNFα,IL-6 and IL-8) and MCP-1 expressions in the trophoblasts were significantly increased, indicating that macrophages can regulate trophoblast inflammatory response. High levels of TNFα,IL-6 and IL-8 in the maternal-fetal interface would be expected to stimulate local inflammatory response which may interfere with embryo developments and survival [34, 35]. Elevated MCP-1 can increase the recruitment of inflammatory cells including monocyte/macrophage into the interface leading to the aggravation of the local inflammatory reaction [18]. In addition, macrphage inflammasome activation-mediated high level IL-1β production also aggravate pro-inflammatory cytokine–mediated inflammation [32, 33], which will not benefit embryo development and pregnancy maintenance. The IL-1β-mediated inflammation might be one of the causes of the dysgenesis and abortion induced by LM infection in humans and animals during gestation period [36, 37]. This study will open a new route for exploring the mechanism of LM-induced spontaneous abortion.

## Conclusion

LM-induced pregnancy failure is associated with macrophage inflammasome activation. LM-induced mouse macrophage inflammasome activations are mainly related to LLO-induced NLRP3 inflammasome activation. The inflammasome-mediated macrophage IL-1β production and IL-1β-induced trophoblast pro-inflammatory cytokine overproduction play crucial roles in LM-induced pregnancy failure.

## Abbreviations

LM: li*steria monocytogenes*
Δ*hly* LM: LLO-deficient LM
LLO: listeriolysin O
PRRs: pattern recognition receptors
TLRs: TOLL-like receptors
AIM2: absent in melanoma-2
NOD: nucleotide-binding oligomerization domain-containing protein
NLRs: NOD-like receptors
NLRP3: NACHT-, LRR- and PYD-containing protein 3
NLRC4: NLR family CARD domain-containing protein 4
RIG-I: retinoic acid inducible gene i
RLRs: RIG-I-like receptors
ALRs: AIM2-like receptors
ROS: reactive oxygen species
ESR: embryo survival rates
MCP-1: monocyte chemotactic protein-1
IRF: interferon regulatory factor
